# Sequence variants in the *PTCH1* gene associate with spine bone mineral density and osteoporotic fractures

**DOI:** 10.1038/ncomms10129

**Published:** 2016-01-06

**Authors:** Unnur Styrkarsdottir, Gudmar Thorleifsson, Sigurjon A. Gudjonsson, Asgeir Sigurdsson, Jacqueline R. Center, Seung Hun Lee, Tuan V. Nguyen, Timothy C.Y. Kwok, Jenny S.W. Lee, Suzanne C. Ho, Jean Woo, Ping-C. Leung, Beom-Jun Kim, Thorunn Rafnar, Lambertus A. Kiemeney, Thorvaldur Ingvarsson, Jung-Min Koh, Nelson L.S. Tang, John A. Eisman, Claus Christiansen, Gunnar Sigurdsson, Unnur Thorsteinsdottir, Kari Stefansson

**Affiliations:** 1 deCODE genetics/Amgen, 101 Reykjavik, Iceland; 2 Osteoporosis and Bone Biology, Garvan Institute of Medical Research, Sydney, New South Wales 2010, Australia; 3 School of Medicine Sydney, University of Notre Dame Australia, Sydney, New South Wales 2010, Australia; 4 Clinical School, St Vincent's Hospital, Sydney, New South Wales 2010, Australia; 5 Faculty of Medicine, University of New South Wales (UNSW), Sydney, New South Wales 2010, Australia; 6 Division of Endocrinology and Metabolism, Asan Medical Center, University of Ulsan College of Medicine, 138-736 Seoul, South Korea; 7 Centre for Health Technologies, University of Technology, Sydney, New South Wales 2010, Australia; 8 Department of Medicine and Therapeutics, Faculty of Medicine, The Chinese University of Hong Kong, Hong Kong, China; 9 JC School of Public Health and Primary Care, Faculty of Medicine, The Chinese University of Hong Kong, Hong Kong, China; 10 Jockey Club Centre for Osteoporosis Care and Control, Faculty of Medicine, The Chinese University of Hong Kong, Hong Kong, China; 11 Radboud University Medical Center, Radboud Institute for Health Sciences, 6500 HB Nijmegen, The Netherlands; 12 Department of Orthopedic Surgery, Akureyri Hospital, 603 Akureyri, Iceland; 13 Institution of Health Science, University of Akureyri, 603 Akureyri, Iceland; 14 Department of Chemical Pathology, and Laboratory for Genetics of Disease Susceptibility, Li Ka Shing Institute of Health Sciences, Faculty of Medicine, The Chinese University of Hong Kong, Hong Kong, China; 15 Clinical Translation and Advanced Education, Garvan Institute of Medical Research, Sydney, New South Wales 2010, Australia; 16 Nordic Bioscience A/S, 2730 Herlev, Denmark; 17 Department of Endocrinology and Metabolism, Landspitali, The National University Hospital of Iceland, 101 Reykjavik, Iceland; 18 Faculty of Medicine, University of Iceland, 101 Reykjavik, Iceland

## Abstract

Bone mineral density (BMD) is a measure of osteoporosis and is useful in evaluating the risk of fracture. In a genome-wide association study of BMD among 20,100 Icelanders, with follow-up in 10,091 subjects of European and East-Asian descent, we found a new BMD locus that harbours the *PTCH1* gene, represented by rs28377268 (freq. 11.4–22.6%) that associates with reduced spine BMD (*P*=1.0 × 10^−11^, *β*=−0.09). We also identified a new spine BMD signal in *RSPO3*, rs577721086 (freq. 6.8%), that associates with increased spine BMD (*P*=6.6 × 10^−10^, *β*=0.14). Importantly, both variants associate with osteoporotic fractures and affect expression of the *PTCH1* and *RSPO3* genes that is in line with their influence on BMD and known biological function of these genes. Additional new BMD signals were also found at the *AXIN1* and *SOST* loci and a new lead SNP at the *EN1* locus.

## Abstract (web summary)

Bone mineral density (BMD) is the best predictor of osteoporotic fracture risk. Here, the authors perform a genome wide association study in Icelanders and people of European and East-Asian descent, and identify a new allele in intron 15 of the *PTCH1* gene that associates with reduced BMD.

## Graphical abstract

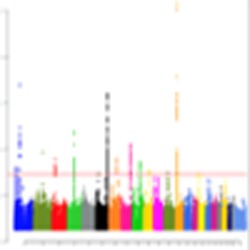

Osteoporosis is a common disease and a major public health problem worldwide with over 9 million osteoporosis-related fractures occurring per year^1^ and associated morbidity and mortality. Osteoporosis is characterized by low bone mineral density (BMD), microarchitectural deterioration of bone tissue and susceptibility to fractures. BMD is the single best predictor of osteoporotic fractures^2^,^3^ and is a valuable tool in evaluating the risk of fractures. There is abundant evidence for a genetic contribution to variation in BMD with heritability estimates between 0.6 and 0.8 (ref. 4). Clearly, environmental and medical factors also influence BMD.

Genome-wide association (GWA) studies (GWAS) of common sequence variants in large sample sets have in recent years lead to the discovery of numerous common sequence variants that associate with variation in BMD and predispose to osteoporosis^5^,^6^,^7^,^8^,^9^,^10^,^11^,^12^,^13^,^14^. The most recent large-scale meta-analysis found 64 independent BMD association signals at 56 loci to meet the criteria of significance^13^. Fourteen of these signals also associate with osteoporotic fractures (*P*<5 × 10^−4^), underscoring the complex relationship between BMD and fracture risk. These established associations are with common sequence variants of small effect on BMD. Many more common sequence variants of similar and smaller effects are expected to be found in future GWAS of larger sample sizes. Whole-genome sequencing (WGS) offers an opportunity to identify and test effects of low frequency (<5% and >1%) and rare (<1%) variants on various traits and diseases. Recent whole-genome sequencing efforts have found both low frequency and rare BMD associations with large effects that is, in the *EN1* (ref. 14) gene, by quantitative analysis of BMD, and in the *LGR4* (ref. 15), and *COL1A2* (ref. 16) genes using low BMD as a dichotomous trait.

Many of the established association signals are near or within genes of the WNT/β-catenin signalling pathway, which is considered to be one of the main pathway controlling bone mass. Inactivation of the pathway results in low BMD and osteoporosis while activating mutations lead to high BMD^17^. Established associations are also observed at genes involved in endochondral ossification, mesenchymal stem cell differentiation, and the RANK/RANKL/OPG pathway^13^.

Despite the large number of sequence variants that associate with BMD they only explain about 6% of the variance in BMD^13^. Here we sought to identify additional sequence variants that influence BMD variation through a GWAS that included 21.5 million sequence variants that had been imputed into about 20,000 Icelanders with BMD measurements at the spine and the hip and with a follow-up in 10,091 subjects of European and East-Asian descent. We found a new BMD locus that harbours the *PTCH1* gene, the receptor for the three hedgehog (Hh) morphogens (SHH, IHH and DHH). The minor allele of rs28377268 (freq. 11.4–22.6%) located in intron 15 of *PTCH1* associates with reduced BMD at the spine (*P*=1.0 × 10^−11^, *β*=−0.09) and an increase in osteoporotic fractures (*P*=8.5 × 10^−4^, OR=1.09) and correlates strongly with 10% increased *PTCH1* expression (*P*=8.2 × 10^−10^). These data are consistent with Ptch1 haploinsufficiency (*Ptch1*^*+/−*^) mice that are characterized by an increase in bone mass. We also identified a new spine BMD signal in *RSPO3* (rs577721086, freq. 6.8%) that associates with increased spine BMD (*P*=6.6 × 10^−10^, *β*=0.14) and decreased risk of osteoporotic fractures (*P*=2.0 × 10^−4^, OR=0.86) and correlates strongly with 40% increased expression of the *RSPO3* gene (*P*=1.3 × 10^−17^). Additional new BMD signals were found at the *AXIN1* and *SOST* loci and a new lead single-nucleotide polymorphism (SNP) at the *EN1* loci. Of note is particularly the strong association of rs71382995 (freq. 9.6%) in *SOST* with vertebral fractures (*P*=4.3 × 10^−9^ and OR=0.56).

## Results

### Genome-wide association analysis

To search for sequence variants that associate with BMD we performed a GWAS of variants that were found in the whole-genomes of 2,636 Icelanders^18^. We imputed the identified SNPs and indels by long-range phasing^19^,^20^ into an Icelandic data set genotyped with Illumina SNP chips (104,220 Icelanders) and used Icelandic genealogical information to calculate genotype probabilities of 294,212 close relatives of those genotyped. From this set we selected those with BMD measurements at the spine, 20,132 in total, or BMD measurements at the hip, 20,162 in total. We then examined association between variation in BMD and the 21.5 million sequence variants found through WGS that passed stringent quality control^18^.

**Figure 1:**
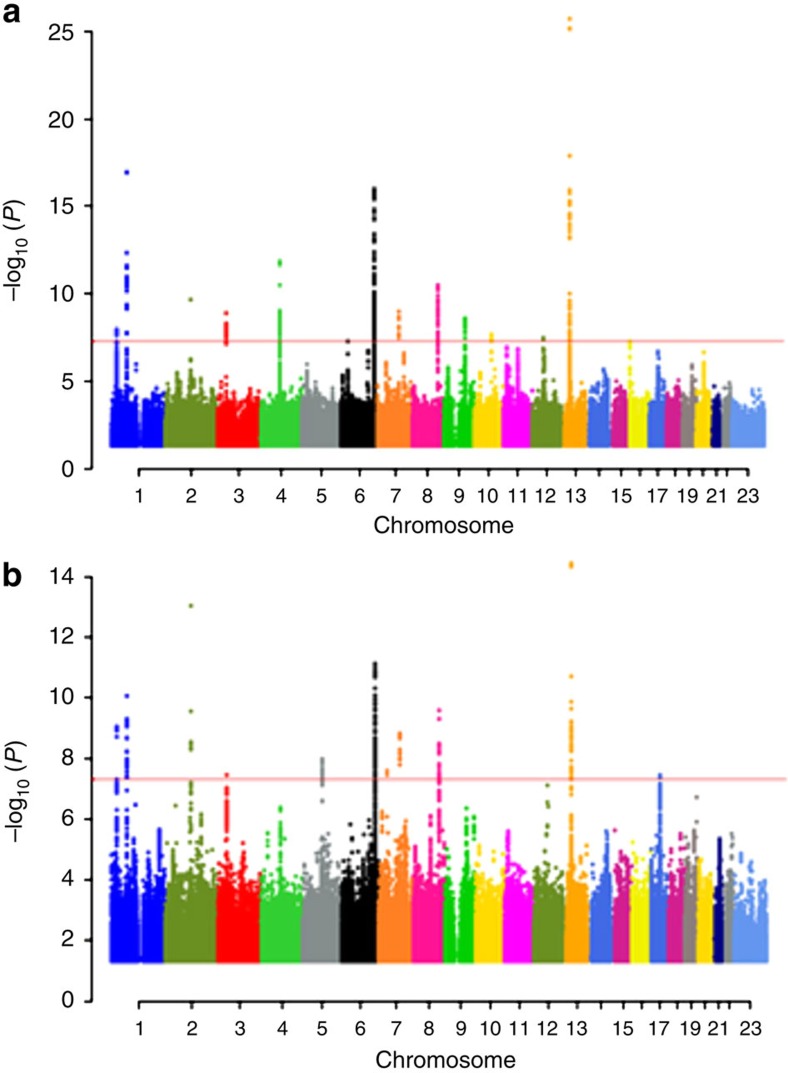
Manhattan plot of discovery genome-wide association study. The *P* values (−log_10_) are plotted against their respective positions on each chromosome. *P*=5 × 10^−8^ is indicated by the horizontal pink line. The plots were created using qqman: an R package for visualizing GWAS results using Q-Q and Manhattan plots^46^. (**a**) Results for spine BMD. The *P* values of the associations are given within brackets at each locus: 1p36.12 (*P*=1.08 × 10^−8^), 1p31.3 (*P*=1.11 × 10^−17^), 2q14.2 (*P*=2.28 × 10^−10^), 3p22.1 (*P*=1.28 × 10^−9^), 4q22.1 (*P*=1.41 × 10^−12^), 6q25.1 (*P*=1.03 × 10^−16^), 7q21.3 (*P*=1.08 × 10^−9^), 8q24.12 (*P*=3.08 × 10^−11^), 9q22.23 (*P*=2.68 × 10^−9^) new locus, 10q22.3 (*P*=2.12 × 10^−8^), 12q13.13 (*P*=3.47 × 10^−8^) and 13q14 (*P*=1.94 × 10^−26^). (**b**) Results for hip BMD. The *P* values of the associations are given within brackets at each locus: 1p36.12 (*P*=8.93 × 10^−10^), 1p31.3 (*P*=8.38 × 10^−11^), 2q14.2 (*P*=9.36 × 10^−14^), 3p22.1 (*P*=3.62 × 10^−8^), 5q14.3 (*P*=2.23 × 10^−9^), 6q25.1 (*P*=7.14 × 10^−12^), 7q21.3 (*P*=1.48 × 10^−9^), 8q24.12 (*P*=2.52 × 10^−10^), 13q14 (*P*=3.54 × 10^−15^), and 17q21.31 (*P*=3.56 × 10^−8^).

**Figure 2:**
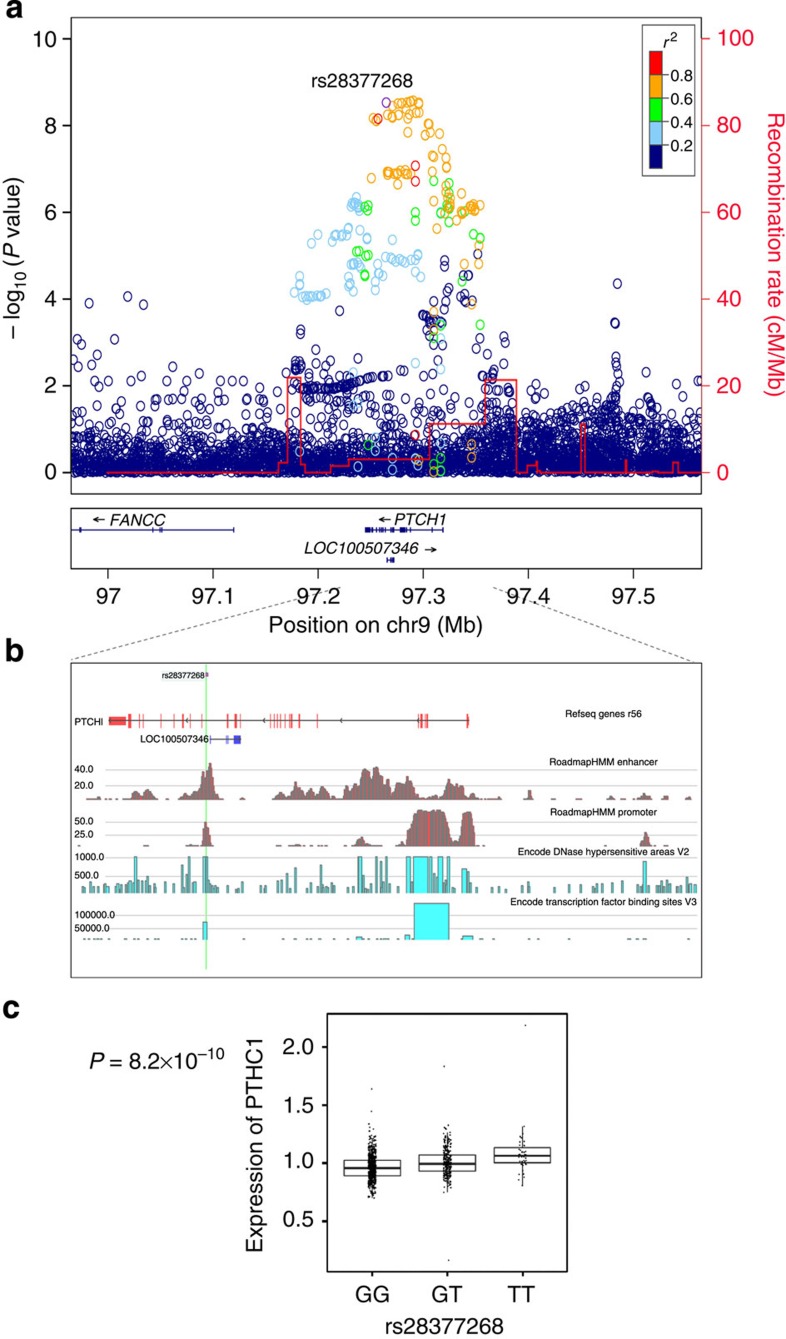
Regional association plot for the 9q22.23 *PTCH1* locus, potential functional elements and genotype-dependent expression. (**a**) Regional association plot for the 9q22.23 *PTCH1* locus. *P* values (−log10) of SNP association with spine BMD in the Icelandic discovery samples are plotted against their positions at the 9q22.23 locus. SNPs are coloured to reflect their linkage disequilibrium (LD) with rs28377268 in the data set. The red line indicates recombination rates, based on the Icelandic recombination map for males and females combined^47^, with the peaks indicating recombination hotspots defining LD blocks in Icelanders. Known genes in the region are shown underneath the plot, taken from the UCSC genes track in the UCSC Genome Browser. All positions are in NCBI Build 36 coordinates. The plot was created using a stand-alone version of LocusZoom software^48^. (**b**) Functional annotation of potential functional elements in the region. Transcription factor binding sites and DNase hypersensitive areas from the ENCODE data is shown^43^,^44^ and enhancer and promoter states from the Roadmap consortium^26^. Location of rs28377268 is indicated by a green vertical line. (**c**) Genotype-dependent gene expression or the *PTCH1* gene in blood samples. The *P* value is derived from regression of the MLR on the carrier status of rs28377268, adjusting for age and sex, and differential cell counts.

In addition to significant associations (*P*<1 × 10^−8^) with variants that have previously been reported to associate with BMD^5^,^6^,^7^,^8^,^9^,^10^,^11^,^12^,^13^,^14^ (Fig. 1) we found a significant association at a new locus on 9q22.23 within the *PTCH1* (patched homologue 1) gene that associates with spine BMD (*P*=2.7 × 10^−9^, *β*=−0.10; Figs 1a and 2a).

### The 9q22.3 *PTCH1* locus

In an attempt to validate the 9q22.23 signal we genotyped the most strongly associated SNP, rs79057214 (freq. 13.3%), in two sample sets of Northern European descent; the Danish PERF (Prospective Epidemiological Risk Factor) study^21^ and the Australian DOES (Dubbo Osteoporosis Epidemiology Study) study^22^, and in two East-Asian populations; KOR-amc (Asan Medical Center) study^23^ from Korea and Chinese samples from Hong Kong^24^. We also genotyped a highly correlated SNP, rs28377268 (freq. 15.7%, *r*^2^=0.79 with rs79057214 in Iceland and *r*^2^=0.86 with rs79057214 in HaploRegv3), as it is predicted to reside in numerous functional elements^25^,^26^ (Fig. 2b and Table 1). Both markers associate with reduced spine BMD in the replication samples with slightly stronger association for rs28377268-T, yielding an overall *P*=2.1 × 10^−11^, *β*=−0.101 for rs79057214-T and *P*=1.0 × 10^−11^, *β*=−0.088 for rs28377268-T, when all samples were analysed together (Table 2). Hence, hereafter we focus on rs28377268. This signal at the *PTCH1* locus also associates with hip BMD, however, the association is weaker (*P*=1.8 × 10^−6^ versus *P*=1.0 × 10^−11^; Supplementary Table 1), demonstrating some skeletal site specificity for this locus. Analysis of other variants at the *PTCH1* locus conditioning on rs28377268 did not identify other independent spine or hip BMD association signals.

The spine BMD-associated markers at this locus reside within and surround the *PTCH1* gene (Fig. 2a). Two exonic markers are among the top markers; a synonymous variant in the *PTCH1* gene, rs1805155 (*P*=1.1 × 10^−7^, *r*^2^=0.74 with rs28377268), and a 3′ variant, rs16909865 (*P*=9.0 × 10^−7^, *r*^2^=0.39 with rs28377268). However, through conditional analysis we show that neither of these putative functional variants account for the rs28377268 association (Supplementary Table 2).

We next analysed the effect of rs28377268-T on osteoporotic fractures in 10,389 cases and 264,522 control samples from Iceland, Denmark, Australia, Korea and China. In accordance with association with reduced BMD, rs28377268-T associates with increased risk of osteoporotic fracture (*P*=0.00085, OR=1.09 for any osteoporotic fracture) (Table 3). When the different fracture sites were analysed independently the largest effect was with vertebral fracture in line with the strongest effect on spine BMD (Supplementary Table 3).

Rs28377268 is in intron 15 of the *PTCH1* gene and based on functional prediction^25^,^26^ rs28377268 overlaps promoter histone marks in 50 organs, enhancer histone marks in 34 organs, sites for binding of 81 proteins and DNase hypersensitivity site in osteoblasts and 101 other cell types and is thus a strong candidate for the causative BMD variant at this locus (Table 1). Since rs28377268 overlaps potential transcriptional regulatory elements we analysed the effect of rs28377268 on expression of *PTCH1* in our available RNA expression data sets from white blood cells and adipose tissue of Icelanders. Although these tissues are not directly relevant to bone, a correlation of the variant with expression may indicate a putative mechanism of the variant. Rs28377268 was strongly correlated with expression of the *PTCH1* gene in blood (*P*=8.2 × 10^−10^), but only weakly in adipose tissue (*P*=0.040). The allele that associates with reduced BMD correlates with 10% increased expression of the gene in white blood cells (Fig. 2c).

A large GWAS study on height has reported four independent height signals at the *PTCH1* locus^27^. Of these four, one (rs4448343) also associates with BMD in Iceland, albeit much weaker than rs28377268, and the association did not remain after adjusting for rs28377268 (Supplementary Table 4). In contrast, rs28377268 associates strongly with height and after adjusting for the four reported height signals we still detect an association (Supplementary Table 4). Rs28377268 thus associates both with BMD and height.

### Additional new BMD signals at known loci

To look for additional new BMD signals we investigated previously reported loci^5^,^6^,^7^,^8^,^9^,^10^,^11^,^12^,^13^,^14^ for independent signals through conditional analyses; conditioning on all reported signals/markers at reported loci. We found new suggestive associations with BMD (*P*<1 × 10^−6^) that were not tagged by reported markers at the 6q22.33 (*RSPO3*) and the 16p13.3 (*AXIN1*) loci that associated with spine BMD, and at the 17q21.31 (*SOST*) locus that associated with both spine and hip BMD. Furthermore, we identified a new lead SNP at the recently reported 2q14.2 (*EN1*)^14^ locus. We genotyped these markers in the samples from Denmark, Australia, Hong Kong and Korea for replication.

### The 6q22.33 *RSPO3* locus

**Figure 3:**
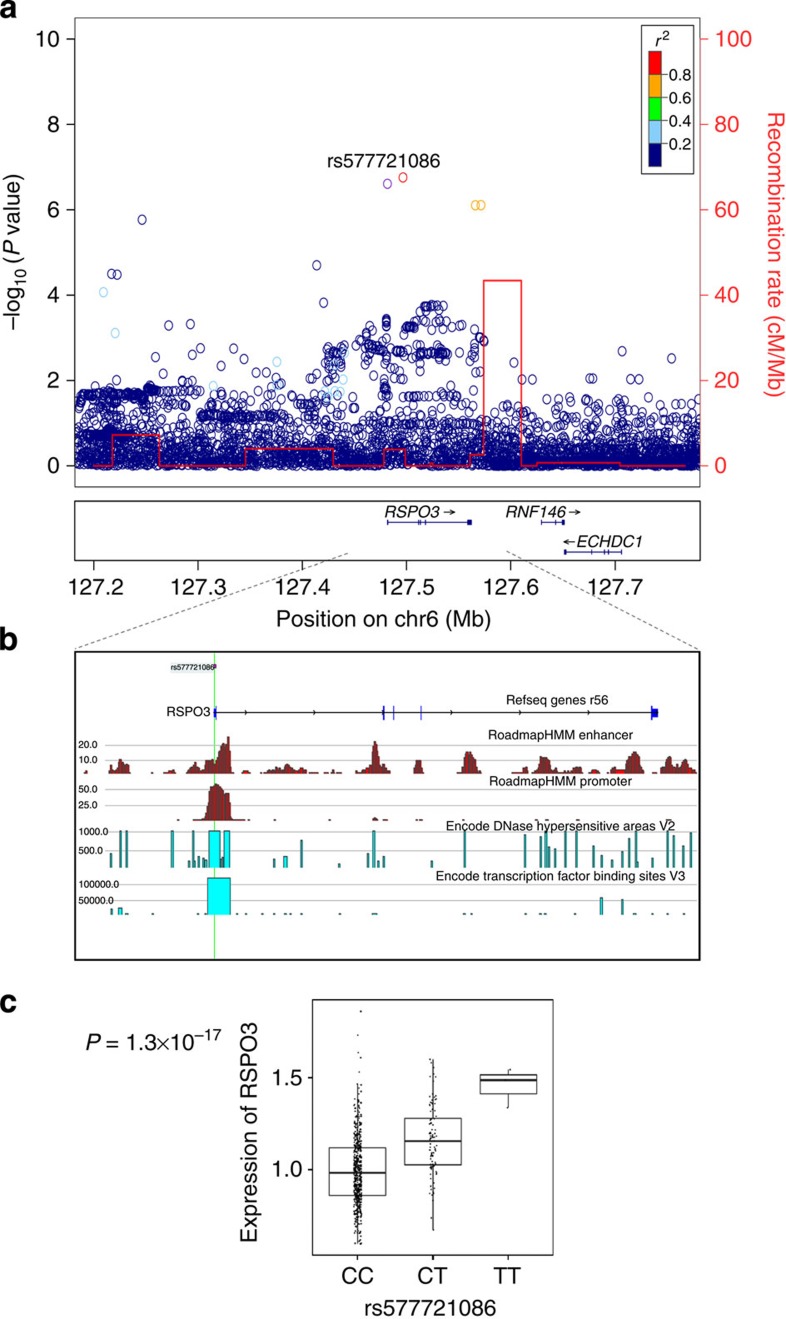
Regional association plot for the 6q22.33 *RSPO3* locus, potential functional elements and genotype-dependent expression. (**a**) Regional association plot for the 6q22.33 *RSPO3* locus. *P* values (−log10) of SNP association with spine BMD in the Icelandic discovery samples are plotted against their positions at the 6q22.33 locus. SNPs are coloured to reflect their linkage disequilibrium (LD) with rs577721086 in the data set. The red line indicates recombination rates, based on the Icelandic recombination map for males and females combined^47^, with the peaks indicating recombination hotspots defining LD blocks in Icelanders. Known genes in the region are shown underneath the plot, taken from the UCSC genes track in the UCSC Genome Browser. All positions are in NCBI Build 36 coordinates. The plot was created using a stand-alone version of LocusZoom software^48^. (**b**) Functional annotation of potential functional elements in the region. Transcription factor binding sites and DNase hypersensitive areas from the ENCODE data is shown^43^,^44^ and enhancer and promoter states from the Roadmap consortium^26^. Location of rs577721086 is indicated by a green vertical line. (**c**) Genotype-dependent gene expression of the *RSPO3* gene. *P* value is derived from regression of the MLR on the carrier status of rs577721086, adjusting for age and sex.

At the *RSPO3* locus (Fig. 3a) we found rs577721086-C (freq.=6.75%) located one base pair upstream of the transcriptional start site that associates with increase in spine BMD independently of previously reported markers^12^,^13^,^28^ (Supplementary Table 5), with *P*=6.6 × 10^−10^, *β*=0.137, when all samples were analysed together (Table 2). This SNP overlaps a DNase hypersensitivity site found in 104 cell types, including osteoblasts (Fig. 3b, Table 1). A considerably weaker association was observed with hip BMD (*P*=0.023, *β*=0.05) indicating skeletal site specificity (Supplementary Table 6). In line with association of rs577721086-C with increase in BMD, we found that rs577721086-C protects against osteoporotic fractures (*P*=0.00020, OR=0.73 for any osteoporotic fracture; Table 3, Supplementary Table 7 for separate skeletal sites).

Analysis of the Icelandic white blood cell and adipose tissue expression data showed that rs577721086-C is correlated with 40% increased expression of *RSPO3* in adipose tissue samples (*P*=3.2 × 10^−17^; Fig. 2c), whereas the gene is not expressed in blood. No other marker in the *RSPO3* region is more strongly associated with *RSPO3* expression.

A common signal at the *RSPO3* locus has been reported to associate with high-density lipoprotein cholesterol (HDL-C) and triglyceride levels^29^ that is not correlated with the spine BMD signal represented by rs577721086-C (Supplementary Table 8). Screening our lipid data we show that rs577721086-C also associates with HDL and triglyceride both in Iceland and samples from the Netherlands (combined analysis, *P*=6.2 × 10^−8^, *β*=−0.07 and *P*=5.0 × 10^−8^, *β*=0.07, respectively). Furthermore, in Iceland the strongest association at this locus with BMD, HDL and triglyceride is represented by rs577721086-C (Supplementary Table 8). These data demonstrate a pleiotropic effect of the *RSPO3* locus on spine BMD and HDL-C/triglyceride levels.

### The 16p13.3 *AXIN1* locus

At the *AXIN1* locus we found a new low frequency signal that was not tagged by the previously reported rs9921222 (ref. 13) SNP (MAF 43%), represented by rs117208012 (freq. 3.5%; Supplementary Table 9). Rs117208012-T associated with spine BMD with *P*=4.6 × 10^−10^, *β*=−0.187 in all sample sets combined (Table 2). Much weaker association was observed with hip BMD and fractures (Supplementary Table 10, Table 3).

### The 17q21.31 *SOST* locus

At the *SOST* locus the new independent signal, rs71382995-A (freq. 9.6%), associates with increase in both hip BMD (*P*=4.8 × 10^−10^, *β*=0.116) and spine BMD (*P*=6.0 × 10^−9^, *β*=0.112) when all samples are analysed together (Table 2, Supplementary Table 11). It is not correlated with previously reported markers at this locus^7^,^13^ and is still nominally associated when conditioned on those markers (Supplementary Table 12). Association with reduced risk of fractures was also observed for this SNP, *P*=5.4 × 10^−7^ and OR=0.78 for all osteoporotic fractures (Table 3), also consistent with the direction of effect on BMD. Of note is particularly the strong association with vertebral fractures with *P*=4.3 × 10^−9^ and OR=0.56 (Supplementary Table 13).

### The 2p14.2 *EN1* locus

Recently, three independent signals at the *EN1* (ref. 14) locus were found to associate with hip BMD (represented by rs55983207, freq. 5.0%) and spine BMD (represented by rs11692564, freq. 1.6% and rs6542457, freq. 5.8%). Both rs55983207 and rs11692564 associate in the Icelandic BMD data sets (*P*=2.8 × 10^−10^ for hip BMD and 6.8 × 10^−7^ for spine BMD, respectively), whereas rs6542457 does not associate (*P*>0.05) (Supplementary Table 14). Using the Icelandic data and conditioning on two of these SNPs (rs55983207 and rs11692564) revealed an additional low-frequency SNP, rs115242848 (freq. 1.2%), that associates with both hip BMD (*P*=9.4 × 10^−14^) and spine BMD (*P*=2.3 × 10^−10^; Table 2, Supplementary Table 15). This SNP tags substantially better the low-frequency signal at this locus previously reported by rs11692564 (ref. 14; Supplementary Table 14), and hence, may be considered a new lead SNP at the locus. Overall, when combining the Icelandic data and that of the replications sets, rs115242848 associated with hip BMD with *P*=8.2 × 10^−13^, *β*=0.348 and with spine BMD with *P*=1.1 × 10^−12^, *β*=0.357 (Table 2) and with osteoporotic fractures (*P*=0.00054, OR=0.61; Table 3, Supplementary Table 16).

## Discussion

We here report a new spine BMD locus harbouring the *PTCH1* gene, and new BMD signals at three previously reported loci (*RSPO3*, *AXIN1* and *SOST*). Importantly, three of these signals, at *PTCH1*, *RSPO3* and *SOST*, associate with the clinically relevant phenotype of osteoporotic fractures in line with their effect on BMD. At both the *PTCH1* and *RSPO3* loci the associated variants are non-coding and affect expression of *PTCH1* and *RSPO3* thus modulating the Hedgehog and the Wnt signalling pathways. Furthermore, we find a new lead low-frequency SNP at the newly reported *EN1* locus^14^.

Rs28377268 in the *PTCH1* gene is moderately common (15.7% in the discovery sample set) yet not detected in previous analyses because it, or an equivalent marker, was not present on the genotyping platforms or the imputed data sets (Hap Map or 1000Gphase1) used in these analyses. The same holds for rs577721086 in the *RSPO3* gene and the variants in *AXIN1* and *SOST* that were not detected in the conditional analyses across the loci reported in Estrada *et al*.^13^ The latter three signals are all under 10% in frequency (6.8%, 3.5% and 9.6%, respectively). The rs115242848 SNP at the *EN1* locus is rarer with a frequency of 1.2%, yet very well imputed in our data set (imputation info=0.995) because of our ability to use long-range phased haplotypes in the imputation. In our data set rs115242848 captures the previously reported signal of rs11692564 (ref. 14) at the *EN1* locus considerably better than rs11692564. This discrepancy between this study and that of Zheng *et al*.^14^ is likely a reflection of difference in imputation quality as variants found in <2% frequency are more difficult to impute in outbred population.

The *PTCH1* gene encodes the receptor (Ptch1) for sonic hedgehog (SHH), indian hedgehog (IHH) and desert hedgehog (DHH). On hedgehog binding, Ptch1 repression of the G-protein-coupled receptor Smoothened (Smo) is released and the hedgehog-signalling cascade is activated. In mice, it has been shown that the hedgehog-Patched1 signalling plays essential roles in many developmental processes, including osteoblastogenesis and chondrocyte differentiation^30^. This pathway is also involved in homeostatic osteoblast activity and in regulation of bone remodelling^31^. Ptch1 haploinsufficiency (*Ptch1*^*+/−*^) mice have increased bone mass as a result of reduced suppression of Smo by Ptch. Furthermore, both systemic interference with Hh signalling^32^ and haploinsufficiency of Gli1 (*Gli1*^*+/−*^), a transcriptional activator induced by Hh signalling, led to decreased bone mass in mice^33^. The correlation we observe in our data set between increased expression of the *PTCH1* gene and association with lowered BMD is consistent with the mouse work; increased levels of PTCH1 represses SMO and Hh signalling which in turn results in reduced bone mass. It is not clear whether this is primarily a developmental effect or one that is relevant through adult life.

We observe a pleiotropic effect of the rs28377268 SNP in the *PTCH1* gene; independent associations with height and with BMD, both traits that reflect aspects of bone biology. The additional height signals that are found at the *PTCH1* locus^27^, however, do not show this pleiotropy in our data set. This difference may mirror differences of spatial and temporal control of *PTCH1* expression and the hedgehog signalling pathway, on one hand bone growth, reflected by association with height and on the other hand bone development/maturation/homeostasis, reflected by association with BMD. The signal tagged by rs28377268 influences both.

Association with BMD near the DHH gene, encoding one of the ligands for PTCH1, has previously been reported^13^. This association was also much stronger for spine BMD than femoral neck BMD, in line with what we observe for the *PTCH1* association in our data set. The importance of the hedgehog signalling pathway in bone development and homeostasis has been well established by functional studies in mice. Its importance in regulation of bone mass in the general human population is now also supported by the association signals at both a hedgehog ligand (DHH) and its receptor (PTCH1).

*RSPO3* is a secreted agonist/enhancer of the Wnt/β-catenin signalling pathway that is considered one of the main regulator of bone mass. *RSPO3* binding to Lgr4 enhances Wnt signal strength and duration^34^. Inactivation of the Wnt/β-catenin pathway results in low BMD and osteoporosis while activating mutations lead to high BMD^17^. The increase in expression of *RSPO3* by rs577721086-C allele, hence, activation of the pathway, is consistent with the association of the SNP with increase in BMD.

Both the *AXIN1* and *SOST* genes encode regulators of the WNT signalling pathway^17^; AXIN1 as a component of the beta-catenin destruction complex and SOST as an extracellular antagonist.

In summary, we report a new spine BMD locus harbouring the *PTCH1* gene, and new BMD signals at three previously reported loci (*RSPO3*, *AXIN1* and *SOST*). Importantly, three of these signals associate with the clinically relevant phenotype of osteoporotic fractures. Of particular interest is the GWS association of our marker in *SOST* with vertebral fractures. At both the *PTCH1* and *RSPO3* loci the associated variants are non-coding that effect expression of *PTCH1* and *RSPO3*, thus modulating the Hedgehog and the Wnt signalling pathways, respectively, both of which have been shown in functional mouse studies to be central to bone development.

## Methods

### Study populations

The Icelandic samples have previously been described in detail^15^. The BMD (DEXA, Hologic QDR4500A) values at the hip (total hip) and lumbar spine were age and weight corrected and standardized in each gender separately. Fracture assessment were as previously described^5^,^7^,^15^, excluding high-trauma fractures, corticosteroid users, early menopause and fractures of the hands, feet and skull. The control groups were individuals who had not suffered low-trauma fracture. All participants gave informed consent and the study was approved by the Data Protection Commission of Iceland (DPC) and the National Bioethics Committee of Iceland.

The Danish samples are postmenopausal women in the age range 55–86 years, taking part in the Prospective Epidemiological Risk Factor (PERF study)^21^. The study was approved by the Ethics Committee of Copenhagen County and was in accordance with the principles of the Helsinki Declaration. The Australian samples were derived from the Dubbo Osteoporosis Epidemiology Study (DOES)^22^, including subjects in the age range 60–99 years. All are of Caucasian ethnicity. The study was approved by the St Vincent's Ethics Review Committee (Sydney). The Chinese Hong Kong samples are comprised of two samples of different sex, the Mr OS and Ms OS studies, aged 65 years and above^35^. The study was approved by the Clinical Research Ethics Committee of the Chinese University of Hong Kong. The Korean samples are postmenopausal women who visited the Osteoporosis Clinic of Asan Medical Center (AMC, Seoul, Korea)^23^. The study was approved by the AMC Ethics Review Committee (Seoul). All participants in these studies provided informed consent, and we obtained approval from all Institutional Review Board to carry out the study.

The Dutch study subjects were recruited within a project entitled ‘Nijmegen Biomedical Study' (NBS). Individuals from the NBS were invited to participate in a study on gene-environment interactions in multifactorial diseases. The details of this study were reported previously^36^. The study protocol of the Nijmegen Biomedical Study was approved by the Institutional Review Board of the Radboud University Medical Center and all study subjects gave written informed consent.

### Genotyping and association analysis

Genotyping and imputation methods and the association analysis method in the Icelandic samples were as described^18^. In short, we sequenced the whole genomes of 2,636 Icelanders using Illumina technology to a mean depth of at least × 10 (median × 20). SNPs and indels were identified and their genotypes called for all samples simultaneously using the Genome Analysis Toolkit (GATK version 2.2-13)^37^. Genotype calls were improved by using information about haplotype sharing, taking advantage of the fact that all the sequenced individuals had also been chip-typed and long-range phased. A total of 19,689,642 SNPs and 1,441,572 indels that met stringent quality criteria were identified in the 2,636 sequenced Icelanders. These variants were then imputed into 104,220 Icelanders who had been genotyped with various Illumina SNP chips and their genotypes phased using long-range phasing^19^,^20^. Genealogical deduction of obligate carrier status of 294,212 untyped relatives of chip-typed individuals further increased the sample size for association analysis and increased the power to detect associations. Individuals who have BMD measurements at the hip or the spine, those who had suffered low-trauma fractures and age- and sex-matched controls were derived from the chip-typed individuals and untyped relatives. Association testing for case–control analysis was performed using logistic regression, and a generalized form of linear regression was used to test for association of quantitative traits.

The whole data set of Icelanders includes a large fraction of the Icelandic population, hence, many of those are related, including those 20,100 in the BMD GWA study. To account for the relatedness and stratification we applied the method of genomic control^38^. The inflation *λ*_g_ in the *χ*^2^-statistic in each GWA was estimated on the basis of a subset of about 300,000 common variants, and *P* values were adjusted by dividing the corresponding *χ*^2^-values by this factor. For the traits reported here, the estimated inflation factors were 1.23 for hip BMD, 1.23 for spine BMD, 1.14 for skull BMD, 1.34 for any osteoporotic fracture, 1.11 for vertebral fractures, 1.20 for hip fractures and 1.27 for forearm fracture, 1.67 for height, 1.40 for triglycerides and 1.58 for HDL cholesterol.

Single-SNP genotyping was carried out on the Centaurus (Nanogen) platform^39^, and by Sanger sequencing (*RSPO3*_ rs577721086). A functional single-SNP genotyping assay could not be made for rs577721086. We, therefore, genotyped a surrogate marker, rs72959041, in the replication samples. We assessed the correlation between rs577721086 and rs72959041 in the replication sample sets by Sanger sequencing approximately 800 individuals from each set; the correlation (*r*^2^) between rs577721086 and rs72959041 was 0.94 in all sets.

### Meta-analysis

Results from multiple case-control groups were combined using a Mantel-Haenszel model^40^ in which groups were allowed to have different population frequencies for alleles and genotypes but were assumed to have common relative risks (a fixed-effect model). Heterogeneity in the effect estimate was tested assuming that the estimated ORs for different groups followed a log-normal distribution and using a likelihood ratio *χ*^2^-test with degrees of freedom equal to the number of groups compared minus one.

### Expression analysis

We investigated the expression of *PTCH1* and *RSPO3* in a data set that included RNA samples from the white blood cells of 1,002 Icelandic individuals and from adipose tissue of 673 individuals^41^. Most of these individuals—973 with white blood cell samples and 646 with adipose tissue samples—had imputed genotypes for the 21.5 million variants identified in whole-genome sequencing. Correlation between expression and the genotypes of the variants was tested by regressing measured MLR (mean log expression ratio) values on the number of copies of the risk-associated allele an individual carried. Effects from age and sex were taken into account by including these variables as explanatory variables. For white blood cells, we also adjusted for differential blood cell count, as these variables correlated strongly with the expression of a large fraction of the genes measured^41^. All *P* values were adjusted for the relatedness of the individuals by simulating genotypes through Icelandic genealogy as previously described^42^. Resulting adjustment factors for the χ^2^-statistic were 1.08 and 1.06 for adipose and whole blood, respectively. The *RSPO3* gene was expressed below reliable detection limits in white blood cells. In adipose tissue, the gene was expressed at high levels. The *PTCH1* gene is expressed at a moderate level in both adipose and in white blood cells.

### Assessment for potential overlap with regulatory regions

To identify the BMD-associated variants that might have regulatory effects we took the strongest signals and for each of the variants and searched for overlaps with known regulatory regions as follows: First we used ENSEMBL to determine whether the variant had been assigned a regulatory region ENSR number. Then we examined the ENCODE data and looked for any evidence of ChIP-Seq transcription factor binding and DNaseI hypersensitivity sites^43^,^44^. We also looked for enhancer and promoter chromatin segmentation states using the 25 state HMM from the Roadmap consortium^26^. Then we looked for correlations between DNaseI hypersensitive sites and local gene expression using results described by Sheffield *et al*.^45^

## Additional information

**How to cite this article:** Styrkarsdottir, U. *et al*. Sequence variants in the *PTCH1* gene associate with spine bone mineral density and osteoporotic fractures. *Nat. Commun.* 7:10129 doi: 10.1038/ncomms10129 (2016).

## Supplementary Material

Supplementary Information Supplementary Tables 1-16

## Figures and Tables

**Table 1:** Overlap of rs28377268 at the 9q22.23—*PTCH1* locus and rs577721086 at the 6q22.33 *RSPO3* locus with potential regulatory regions.

CHR	Marker	ENSR number	ENCODE ChIP-Seq transcription factors bound	ENCODE DNaseI HS site (cell types)	Enhancer Chromatin state roadmap	Promoter Chromatin state roadmap
chr6	rs577721086	ENSR00001226472	22	104	7	71
chr9	rs28377268	ENSR00001309093	81	102	34	50

ENSR number, Ensembl Regulatory feature number, ENCODE, Encyclopedia of DNA Elements, ChIP-Seq, chromatin immunoprecipitation sequencing, DNaseI, Deoxyribonuclease I.

Overlap of genomic position of rs577721086 and rs28377268 with Ensembl ENSR number are shown, as well as number of cell types overlapping transcription factors binding sites as determined by ChIP-Seq and open chromatin state of the chromosome as DNaseI hypersensitivity sites^43^,^44^. Enhancer and promoter chromatin segmentation states using the 25 state HMM from the Roadmap consortium^26^ are also shown.

**Table 2:** Association of new signals with spine BMD and hip BMD.

Region, SNP	EA/OA	Freq. %	Icelandic discovery set		Replication sets			All sets combined	
			*P* value	Effect	*P* value	Effect	*P* value	Effect (95% CI)	*P* het
Spine bone mineral density:			*N*=20,132		*N*=10,092			*N*=30,224	
									
9q22.23—*PTCH1*									
rs28377268	T/G	15.71	3.0 × 10^−9^	−0.102	0.00034	−0.068	1.0 × 10^−11^	−0.088 (−0.112, −0.062)	0.36
									
6q22.33—*RSPO3*									
rs577721086*	C/T	6.75	2.5 × 10^−7^	0.133	0.00066	0.149	6.6 × 10^−10^	0.137 (0.094, 0.181)	0.53
									
16p13.3—*AXIN1*									
rs117208012	T/C	3.49	4.6 × 10^−7^	−0.175	0.00020	−0.223	4.6 × 10^−10^	−0.187 (−0.246, −0.128)	0.77
									
17q21.31—*SOST*									
rs71382995	A/G	9.56	1.9 × 10^−7^	0.115	0.0088	0.101	6.0 × 10^−9^	0.112 (0.074, 0.149)	0.048
									
2q14.2—*EN1*									
rs115242848	T/C	1.22	2.3 × 10^−10^	0.371	0.0011	0.318	1.1 × 10^−12^	0.357 (0.259, 0.455)	0.39
									
Hip bone mineral density:			*N*=20,162		*N*=10,037			*N*=30,199	
									
17q21.31—*SOST*									
rs71382995	A/G	9.56	3.3 × 10^−7^	0.109	0.00029	0.140	4.8 × 10^−10^	0.116 (0.080, 0.153)	0.32
									
2q14.2—*EN1*									
rs115242848	T/C	1.22	9.4 × 10^−14^	0.421	0.14	0.140	8.2 × 10^−13^	0.348 (0.253, 0.444)	0.040

BMD, bone mineral density; Freq., frequency in the Icelandic samples; *N*, total number of individuals in the BMD analyses; SNP, single-nucleotide polymorphism; 95% CI, 95% confidence interval of the effect.

The estimated effects, expressed as standardized values (s.d. above or below the population average) per copy of the SNP allele, and *P* values are derived from a linear regression of the age-, sex- and weight-adjusted BMD values on the SNP status. All *P* values are corrected for relatedness using the method of genomic controls. EA designate the effect allele and OA the other allele. Results are shown for the Icelandic discovery set, the combined replication sets, and the overall results for the discovery and replication samples combined.

^*^A surrogate marker, rs72959041, was used instead of rs577721086 for genotyping the replication samples (*r*^2^=0.94 in all sample sets based on a sequenced subset of all samples) because a functional assay could not be made for rs577721086.

**Table 3:** Association with osteoporotic fractures.

Region, SNP	EA/OA	Freq. %	Icelandic discovery set		Replication sets		All sets combined		
			*P* value	OR	*P* value	OR	*P* value	OR (95% CI)	*P* het
Any osteoporotic fracture:			*N*=7,836/261,563		*N*=1,982/1,790		*N*=10,389/264,522		
									
9q22.23—*PTCH1*									
rs28377268	T/G	15.71	0.0023	1.09	0.17	1.08	8.5 × 10^−4^	1.09 (1.04, 1.15)	0.57
									
6q22.33—*RSPO3*									
rs577721086*	C/T	6.75	0.0093	0.89	0.0012	0.73	2.0 × 10^−4^	0.86 (0.79, 0.93)	0.17
									
16p13.3—*AXIN1*									
rs117208012	T/C	3.49	0.22	1.18	0.072	1.34	0.053	1.17 (1.00, 1.36)	0.62
									
17q21.31—*SOST*									
rs71382995	A/G	9.56	6.3 × 10^−4^	0.81	0.00010	0.71	5.4 × 10^−7^	0.78 (0.71, 0.86)	0.16
									
2q14.2—*EN1*									
rs115242848	T/C	1.22	0.015	0.66	0.011	0.53	5.4 × 10^−4^	0.61 (0.46, 0.81)	0.79

Freq., frequency in the Icelandic samples; *N*, number of fractured cases/and controls; OR, odds ratio; SNP, single-nucleotide polymorphism. 95% CI, 95% confidence interval of the OR.

All *P* values are corrected for relatedness using the method of genomic controls. EA designate the effect allele and OA the other allele. Results are shown for the Icelandic discovery set, the combined replication sets, and the overall results for the discovery and replication samples combined. See Supplementary Tables for association with fractures at different skeletal sites.

^*^A surrogate marker, rs72959041, was used instead of rs577721086 for genotyping the replication samples (*r*^2^=0.94 in all sample sets based on a sequenced subset of all samples) because a functional assay could not be made for rs577721086.
