# Supplementary material for: Sequence variants in the PTCH1 gene associate with spine bone mineral density and osteoporotic fractures
Source: Nat Commun. 2016 Jan 6;7:10129. doi: 10.1038/ncomms10129 (PMC4729819; doi:10.1038/ncomms10129)
Supplement: Supplementary Information — Supplementary Tables 1-16 [file ncomms10129-s1.pdf]

Supplementary Tables:

Supplementary Table 1. Association of rs28377268 [T] in *PTCH1* with hip and spine BMD in the discovery and individual replication samples

Locus / SNP	P value	effect	P value	effect	P value	effect	P value	effect	P value	effect	*P* value	effect	*P* het
**Spine BMD**	**Iceland (20,132)**		**DK (3,715)**		**AUS (1,324)**		**HK (3,668)**		**KOR (1,385)**		**All sets combined (30,224)**		
rs28377268 [T]	3.0E-09	-0.102	3.6E-03	-0.101	0.61	-0.031	0.16	-0.041	0.029	-0.100	1.0E-11	-0.09 (-0.11, -0.06)	0.36
**Hip BMD**	**Iceland (20,162)**		**DK (3,696)**		**AUS (1,279)**		**HK (3,677)**		**KOR (1,385)**		**All sets combined (30,199)**		
rs28377268 [T]	9.4E-07	-0.082	0.057	-0.063	0.87	0.010	0.85	-0.005	0.21	-0.058	1.80E-06	-0.06 (-0.08, -0.04)	0.16

The frequency of rs28377268[T] in the listed populations is: Iceland = 15.71%, Denmark = 13.11%, Australia = 11.4%, Hong Kong = 19.9% and Korea = 22.6%

Supplementary Table 2. Pair-wise correlations and conditional analyses between markers associated with spine BMD at 9q22.23 *PTCH1*

Marker	Allele	Freq.%	β	*P*	rs79057214		rs28377268		rs1805155		rs16909865		rs1805155 & rs16909865
					r^2	P_{adj}	r^2	P_{adj}	r^2	P_{adj}	r^2	P_{adj}	P_{adj}
**rs79057214**	T	13.13	-0.111	2.7E-09	-	-	0.79	0.16	0.95	0.0020	0.51	4.8E-04	0.0068
**rs28377268**	T	15.71	-0.102	3.0E-09	0.79	0.18	-	-	0.74	0.0075	0.39	2.8E-04	0.014
**rs1805155**	G	14.03	-0.096	1.1E-07	0.95	0.13	0.74	0.72	-	-	0.48	0.0075	-
**rs16909865**	G	11.03	-0.099	9.0E-07	0.51	0.34	0.39	0.14	0.48	0.078	-	-	-

Association with variations in spine BMD for markers at 9q22.23. The table includes the the allele, frequency (Freq%), effect (β), and P-value for the test of association. Also included are correlations between SNPs and an adjusted P-value, *P*adj, corresponding to test of association done adjusting for the observed association with the other variant. All *P* values have been adjusted for relatedness using the method of genomic control.

Supplementary table 3: Association of rs28377268 [T] in *PTCH1* with osteoporotic fractures at different skeletal sites in the discovery and individual replication samples

P value	OR	*P* value	OR	*P* value	OR	*P* value	OR	*P* value	OR	*P* value	OR	*P* het
All fractures												
**Ice (7,836/261,563)**		**DK (1,500/1,284)**		**AUS (482/506)**		**HK (400/826)**		**KOR (171/343)**		**All sets combined (10,389/264,522)**		
0.0023	1.09	0.13	1.13	0.31	0.86	0.23	1.14	0.81	1.05	8.5E-04	1.09 (1.04, 1.15)	0.73
Vertebral fracture												
**Ice (263/176,455)**		**DK (824/1,284)**		**AUS (189/506)**		**HK**		**KOR (106/343)**		**All sets combined (1,382/178,588)**		
0.26	1.15	0.21	1.13	0.20	1.26	NA	NA	0.37	0.84	0.063	1.13(0.99,1.33)	0.76
Hip fracture												
**Ice (6,239/115,587)**		**DK (79/1,284)**		**AUS (126/506)**		**HK**		**KOR**		**All sets combined (6,444/117,377)**		
0.015	1.08	0.53	1.15	0.65	0.88	NA	NA	NA	NA	0.15	1.08(1.02,1.15)	0.74
Forearm fracture												
**Ice (1,102/196,718)**		**DK (569/1,284)**		**AUS (113/506)**		**HK**		**KOR**		**All sets combined (1,784/198,508)**		
0.013	1.18	0.62	1.06	0.24	0.74	NA	NA	NA	NA	0.035	1.12 (1.01, 1.25)	0.19

The frequency of rs28377268[T] in the listed populations is: Iceland = 15.71%, Denmark = 13.11%, Australia = 11.4%, Hong Kong = 19.9% and Korea = 22.6%

Supplementary Table 4. Pair-wise correlations and association with height and BMD of previously reported markers for height and rs28377268 for BMD at 9q22.23

						GIANT Height meta-analysis[†]		Icelandic dataset								
								Height			Spine BMD			Hip BMD		
SNP	Chr	EA	OA	EAF[*]	r^{2*}	Beta	P-value	Beta	P	P**	Beta	P	P***	Beta	P	P***
**Published heigth variants**																
rs4448343	9	G	A	0.382	0.15	0.035	4.6E-30	0.033	4.8E-06	-	-0.046	0.0004	0.91	-0.046	0.0002	0.057
rs1329393	9	T	C	0.177	<0.01	0.034	2.4E-13	0.037	5.2E-05	-	-0.021	0.20	0.27	-0.025	0.12	0.15
rs817300	9	A	G	0.061	<0.01	-0.085	4.3E-34	-0.079	4.9E-08	-	0.016	0.56	0.70	0.039	0.13	0.18
rs10990303	9	T	C	0.213	0.02	0.036	4.4E-24	0.039	4.8E-06	-	0.003	0.86	0.34	-0.018	0.23	0.58
**BMD variant**																
rs28377268	9	T	G	0.157	-	-	-	0.047	7.5E-07	0.002	-0.109	3.0E-09	-	-0.082	9.4E-07	-

* EAF and r2 with rs28377268 in the Icelandic dataset

** P-value adjusted for rs4448343, rs1329393, rs817300 and rs10990303

*** P-value adjusted for rs28377268

[†]Markers reported in Wood *et al*, 2014, Nat Genet

Supplementary Table 5. Pair-wise correlations and conditional analyses between markers associated with spine BMD at 6q22.33 *RSPO3*

					rs577721086		rs13204965		rs7741021		rs13204965 & rs7741021
**Marker**	**Allele**	**Freq.%**	**β**	***P***	r^2	P_{adj}	r^2	P_{adj}	r^2	P_{adj}	P_{adj}
rs577721086*	C	6.75	0.133	2.5E-07	-	-	0.025	8.8E-07	0.068	8.3E-06	1.7E-04
rs13204965†	C	26.2	-0.028	0.047	0.025	0.22	-	-	0.113	0.0015	-
rs7741021††	A	49.9	-0.040	0.0015	0.068	0.067	0.113	5.6E-05	-	-	-

Association with variations in Spine BMD at 6q22.33. The table includes the allele, frequency (Freq%), effect (β), and *P*-value for the test of association. Also included are correlations between SNPs and an adjusted *P*-value (*P* adj) corresponding to test of association done adjusting for the observed association with the other variant. All *P* values have been adjusted for relatedness using the method of genomic control.

†Marker reported in Duncan *et al*, 2011, PLoS Genet

†† Marker reported in Moayyeri *et al*, 2014, Hum Mol Genet

*A surrogate marker, rs72959041, was used instead of rs577721086 for genotyping the replication samples (r^2 = 0.94 in all sample sets) because a functional assay could not be made for rs577721086.

Supplementary Table 6. Association of rs577721086 [T]* in *RSOP3* with hip and spine BMD in the discovery and individual replication samples

Locus / SNP	P value	effect	P value	effect	P value	effect	P value	effect	P value	effect	*P* value	effect	*P* het
**Spine BMD**	**Iceland (20,132)**		**DK (3,715)**		**AUS (1,324)**		**HK (3,668)**		**KOR (1,385)**		**All sets combined (30,224)**		
rs577721086 [C]*	2.5E-07	0.133	0.012	0.124	0.011	0.240	NA	NA	NA	NA	6.6E-10	0.14 (0.09, 0.18)	0.53
**Hip BMD**	**Iceland (20,162)**		**DK (3,696)**		**AUS (1,279)**		**HK (3,677)**		**KOR (1,385)**		**All sets combined (30,199)**		
rs577721086 [C]*	0.020	0.058	0.66	-0.021	0.042	0.192	NA	NA	NA	NA	0.023	0.05 (0.01, 0.09)	0.10

*A surrogate marker, rs72959041, was used instead of rs577721086 for genotyping the replication samples (r2 = 0.94 in all sample sets) because a functional assay could not be made for rs577721086. The frequency of rs577721086 [T] in the listed populations is: Iceland = 6.75%, Denmark = 6.70%, Australia = 4.82%, and Korea = not polymorphic. In Hong Kong only one individual was found with the rs577721086 [T] genotype.

Supplementary table 7: Association of rs577721086 [C]* in RSOP3 with osteoporotic fractures at different skeletal sites in the discovery and individual replication samples

P value	OR	*P* value	OR	*P* value	OR	*P* value	OR	*P* value	OR	*P* value	OR	*P* het
All fractures												
**Ice (7,836/261,563)**		**DK (1,500/1,284)**		**AUS (482/506)**		**HK (400/826)**		**KOR (171/343)**		**All sets combined (10,389/264,522)**		
0.0093	0.89	0.0042	0.73	0.12	0.72	NA	NA	NA	NA	2.0E-04	0.86 (0.79, 0.93)	0.17
Vertebral fracture												
**Ice (263/176,455)**		**DK (824/1,284)**		**AUS (189/506)**		**HK**		**KOR (106/343)**		**All sets combined (1,382/178,588)**		
0.032	0.63	0.0091	0.71	0.80	1.06	NA	NA	NA	NA	0.0032	0.74 (0.61, 0.90)	0.25
Hip fracture												
**Ice (6,239/115,587)**		**DK (79/1,284)**		**AUS (126/506)**		**HK**		**KOR**		**All sets combined (6,444/117,377)**		
0.44	0.96	0.22	0.61	0.014	0.34	NA	NA	NA	NA	0.23	0.94 (0.85, 1.04)	0.036
Forearm fracture												
**Ice (1,102/196,718)**		**DK (569/1,284)**		**AUS (113/506)**		**HK**		**KOR**		**All sets combined (1,784/198,508)**		
0.040	0.76	0.029	0.72	0.067	0.45	NA	NA	NA	NA	9.2E-04	0.72 (0.60, 0.88)	0.51

*A surrogate marker, rs72959041, was used instead of rs577721086 for genotyping the replication samples (r2 = 0.94 in all sample sets) because a functional assay could not be made for rs577721086. The frequency of rs577721086 [T] in the listed populations is: Iceland = 6.75%, Denmark = 6.70%, Australia = 4.82%, and Korea = not polymorphic. In Hong Kong only one individual was found with the rs577721086 [T] genotype.

Supplementary Table 8: Association analysis of rs577721086[C] and reported lipid and BMD SNPs with HDL, triglyceride and BMD

**a) Triglycerides**					**rs577721086**		**rs1936800**	
**Marker**	**Allele**	**Freq.%**	**β**	***P***	**r^2**	**P_{adj}**	**r^2**	**P_{adj}**
**Iceland**								
rs577721086*	C	6.75	0.065	3.1E-07	-	-	0.051	9.3E-06
rs1936800[†]	C	42.02	-0.022	5.0E-04	0.051	0.018	-	-
rs13204965[††]	C	26.20	0.003	0.68	0.025	0.21	0.073	0.62
rs7741021[†††]	C	49.87	-0.015	0.014	0.068	0.27	0.56	0.88
**The Netherland**								
rs577721086*	C	6.16	0.076	0.058	-	-	-	-
**Iceland and The Netherland**								
rs577721086*	C	6.16	0.07	5.0×10^{-8}	-	-	-	-

**b) HDL Cholesterol**					**rs577721086**		**rs1936800**	
**Marker**	**Allele**	**Freq.%**	**β**	***P***	**r^2**	**P_{adj}**	**r^2**	**P_{adj}**
rs577721086*	C	6.75	-0.065	1.4E-06	-	-	0.051	7.2E-05
rs1936800[†]	C	42.02	0.028	3.4E-05	0.051	0.0019	-	-
rs13204965[††]	C	26.20	-0.004	0.63	0.025	0.21	0.073	0.63
rs7741021[†††]	C	49.87	0.028	2.9E-05	0.068	0.0029	0.56	0.36
**The Netherland**								
rs577721086*	C	6.16	-0.105	0.0087	-	-	-	-
**Iceland and The Netherland**								
rs577721086*	C	6.16	-0.07	6.2×10^{-8}	-	-	-	-

**c) Spine BMD**								
**Marker**	**Allele**	**Freq.%**	**β**	***P***	**r^2**	**P_{adj}**	**r^2**	**P_{adj}**
rs577721086*	C	6.75	0.133	2.5E-07	-	-	0.051	5.2E-06
rs1936800[†]	C	42.02	-0.039	0.0021	0.051	0.057	-	-
rs13204965[††]	C	26.20	-0.028	0.047	0.025	0.22	0.073	0.0039
rs7741021[†††]	C	49.87	-0.04	0.0015	0.068	0.067	0.56	0.18

Association with variations in a) Triglycerides, b) HDL cholesterol and c) spine BMD. The table includes the allele, frequency (Freq%), effect (β), and *P*-value for the test of association. Also included are correlations between SNPs and an adjusted *P*-value (*P*adj) corresponding to test of association done adjusting for the observed association with the other variant. All *P* values have been adjusted for relatedness using the method of genomic control. *A surrogate marker, rs72959041, was used instead of rs577721086 for genotyping the Dutch replication samples (r2 = 0.94 in all sample sets) because a functional assay could not be made for rs577721086.

[†]Marker reported by Global Lipids Genetics Consortium, 2013, Nat Gen

[††]Marker reported in Duncan *et al*, 2011, PLoS Genet to be associated with hip BMD

[†††]Marker reported in Moayyeri *et al*, 2014, Hum Mol Genet to be associated with heel ultrasound

Supplementary Table 9. Pair-wise correlations and conditional analyses of BMD associations at the 16p13.3 *Axin1* locus

					rs9921222		rs117208012	
**Marker**	**Allele**	**Freq.%**	**β**	***P***	r^2	P_{adj}	r^2	P_{adj}
rs9921222*	C	42.7	0.070	5.3E-08	-	-	0.025	3.3E-06
rs117208012	T	3.49	−0.175	4.6E-07	0.025	3.0E-05	-	-

*Marker reported in Estrada *et a*l, 2012, Nat Genet.

Supplementary Table 10. Association of rs117208012 [T] in *AXIN1* with hip and spine BMD in the discovery and individual replication samples

Locus / SNP	P value	effect	P value	effect	P value	effect	P value	effect	P value	effect	*P* value	effect	*P* het
**Spine BMD**	**Iceland (20,132)**		**DK (3,715)**		**AUS (1,324)**		**HK (3,668)**		**KOR (1,385)**		**All sets combined (30,224)**		
rs117208012 [T]	4.6E-07	−0.175	7.2E-04	−0.229	0.12	−0.203	NA	NA	NA	NA	4.6E-10	−0.187 (−0.246, −0.128)	0.77
**Hip BMD**	**Iceland (20,162)**		**DK (3,696)**		**AUS (1,279)**		**HK (3,677)**		**KOR (1,385)**		**All sets combined (30,199)**		
rs117208012 [T]	0.0026	−0.101	0.018	−0.157	0.84	−0.026	NA	NA	NA	NA	0.00022	-0.108 (-0.165, -0.051)	0.61

The frequency of rs117208012 [T] in the listed populations is: Iceland = 3.49%, Denmark = 3.24%, Australia = 2.21%, Hong Kong = non polymorphic.

Supplementary Table 11. Association of rs71382995 [A] in *SOST* with hip and spine BMD in the discovery and individual replication samples

Locus / SNP	P value	effect	P value	effect	P value	effect	P value	effect	P value	effect	*P* value	effect	*P* het
**Spine BMD**	**Iceland (20,132)**		**DK (3,715)**		**AUS (1,324)**		**HK (3,668)**		**KOR (1,385)**		**All sets combined (30,224)**		
rs71382995 [A]	1.9E-07	0.115	0.054	0.085	0.12	0.127	0.0034	1.68	NA	NA	4.8E-10	0.116 (0.080, 0.153)	0.32
**Hip BMD**	**Iceland (20,162)**		**DK (3,696)**		**AUS (1,279)**		**HK (3,677)**		**KOR (1,385)**		**All sets combined (30,199)**		
rs71382995 [A]	3.3E-07	0.109	0.0074	0.117	0.014	0.200	0.081	1.00	NA	NA	6.0E-09	0.112 (0.074, 0.149)	0.048

The frequency of rs71382995 [A] in the listed populations is: Iceland = 9.56%, Denmark = 8.66%, Australia = 6.22%, Hong Kong = 0.058% and Korea = not polymorphic.

Supplementary Table 12. Pair-wise correlations and conditional analyses of BMD associations at the 17q21.31 *SOST* locus

**a) Spine BMD**					**rs7220711**		**rs1513670**		**rs1107748**		**rs71382995**		
**Marker**	**Allele**	**Freq.%**	**β**	***P***	r^2	P_{adj}	r^2	P_{adj}	r^2	P_{adj}	r^2	P_{adj}	P_{adj}**
rs7220711*	G	36.8	0.063	2.9E-06	-	-	0.24	0.0025	0.83	0.092	0.17	0.0061	0.39
rs1513670*	T	36.8	−0.055	3.2E-05	0.24	0.032	-	-	0.27	0.025	0.055	0.0021	0.040
rs1107748*	C	36.2	0.058	1.2E-05	0.83	0.77	0.27	0.0088	-	-	0.15	0.010	0.92
rs71382995	A	9.56	0.115	2.0E-07	0.17	3.6E-04	0.055	1.2E-05	0.15	1.4E-04	-	-	4.3E-04

**a) Hip BMD**					**rs7220711**		**rs1513670**		**rs1107748**		**rs71382995**		
**Marker**	**Allele**	**Freq.%**	**β**	***P***	r^2	P_{adj}	r^2	P_{adj}	r^2	P_{adj}	r^2	P_{adj}	P_{adj}**
rs7220711*	G	36.8	0.071	3.7E-08	-	-	0.24	3.7E-04	0.83	0.012	0.17	2.1E-04	0.084
rs1513670*	T	36.8	−0.062	9.8E-07	0.24	0.012	-	-	0.27	0.0053	0.055	1.2E-04	0.011
rs1107748*	C	36.2	0.063	9.5E-07	0.83	0.82	0.27	0.0051	-	-	0.15	0.0015	0.48
rs71382995	A	9.56	0.109	3.3E-07	0.17	0.0021	0.055	3.9E-05	0.15	4.9E-04	-	-	0.0025

*Marker reported in Styrkarsdottir *et al*, 2009, Nat Genet. Rs7220711 and rs4792909 (Estrada *et al*, 2012, Nat Genet) are equivalent markers. Rs7220711 and rs1107748 were found to be independent in a previous study.

***P* value adjusted for all the other markers

Supplementary table 13: Association of rs71382995 [A] in *SOST* with osteoporotic fractures at different skeletal sites in the discovery and individual replication samples

P value	OR	*P* value	OR	*P* value	OR	*P* value	OR	*P* value	OR	*P* value	OR	*P* het
All fractures												
**Ice (7,836/261,563)**		**DK (1,500/1,284)**		**AUS (482/506)**		**HK (400/826)**		**KOR (171/343)**		**All sets combined (10,389/264,522)**		
6.3E-04	0.81	4.1E-05	0.66	0.58	0.90	NA	NA	NA	NA	5.4E-07	0.78 (0.71, 0.86)	0.16
Vertebral fracture												
**Ice (263/176,455)**		**DK (824/1,284)**		**AUS (189/506)**		**HK**		**KOR (106/343)**		**All sets combined (1,382/178,588)**		
1.5E-05	0.42	2.7E-05	0.60	0.26	0.72	NA	NA	NA	NA	4.3E-09	0.56 (0.46, 0.68)	0.21
Hip fracture												
**Ice (6,239/115,587)**		**DK (79/1,284)**		**AUS (126/506)**		**HK**		**KOR**		**All sets combined (6,444/117,377)**		
0.13	0.94	0.0040	0.31	0.47	0.76	NA	NA	NA	NA	0.063	0.93 (0.86, 1.00)	0.022
Forearm fracture												
**Ice (1,102/196,718)**		**DK (569/1,284)**		**AUS (113/506)**		**HK**		**KOR**		**All sets combined (1,784/198,508)**		
0.032	0.83	2.7E-04	0.61	0.39	1.26	NA	NA	NA	NA	6.4E-04	0.79 (0.68, 0.90)	0.03

The frequency of rs71382995 [A] in the listed populations is: Iceland = 9.56%, Denmark = 8.66%, Australia = 6.22%, Hong Kong = 0.058% and Korea = not polymorphic.

Supplementary Table 14. Pair-wise correlations and conditional analyses of BMD associations at 2q14.2 *EN1*

a) Spine BMD					rs55983207		rs11692564		rs188303909		rs115242848		
**Marker**	**Allele**	**Freq.%**	**β**	***P***	r^2	P_{adj}	r^2	P_{adj}	r^2	P_{adj}	r^2	P_{adj}	P_{adj}**
rs55983207*	C	4.97	0.045	0.13	-	-	0.0014	0.088	0.0025	0.088	7.0E-04	0.086	0.078
rs11692564*	T	2.16	0.219	6.8E-07	0.0014	4.9E-07	-	-	0.55	0.022	0.36	0.32	0.99
rs188303909*	T	3.44	0.162	6.0E-06	0.0025	4.3E-06	0.55	0.30	-	-	0.19	0.15	0.28
rs115242848	T	1.22	0.371	2.3E-10	7.0E-04	1.6E-10	0.36	4.7E-05	0.19	3.0E-06	-	-	4.3E-05

a) Hip BMD					rs55983207		rs11692564		rs188303909		rs115242848		
**Marker**	**Allele**	**Freq. %**	**β**	***P***	r^2	P_{adj}	r^2	P_{adj}	r^2	P_{adj}	r^2	P_{adj}	P_{adj}**
rs55983207*	C	4.97	0.183	2.8E-10	-	-	0.0014	5.3E-11	0.0025	5.6E-11	7.0E-04	4.6E-11	3.2E-11
rs11692564*	T	2.16	0.250	4.3E-09	0.0014	8.2E-10	-	-	0.55	0.0048	0.36	0.21	0.86
rs188303909*	T	3.44	0.182	1.5E-07	0.0025	2.9E-08	0.55	0.29	-	-	0.19	0.10	0.22
rs115242848	T	1.22	0.421	9.4E-14	7.0E-04	1.6E-14	0.36	2.00E-06	0.19	3.2E-08	-	-	1.3E-06

*Marker reported in Zheng *et al*, 2015, Nature ** *P* value adjusted for the three other markers

Supplementary Table 15. Association of rs115242848 [T] in *EN1* with hip and spine BMD in the discovery and individual replication samples

Locus / SNP	P value	effect	P value	effect	P value	effect	P value	effect	P value	effect	*P* value	effect	*P* het
**Spine BMD**	**Iceland (20,132)**		**DK (3,715)**		**AUS (1,324)**		**HK (3,668)**		**KOR (1,385)**		**All sets combined (30,224)**		
rs115242848 [T]	2.3E-10	0.371	0.021	0.253	0.0081	0.558	NA	NA	NA	NA	1.1E-12	0.348 (0.253, 0.444)	0.040
**Hip BMD**	**Iceland (20,162)**		**DK (3,696)**		**AUS (1,279)**		**HK (3,677)**		**KOR (1,385)**		**All sets combined (30,199)**		
rs115242848 [T]	9.4E-14	0.421	0.22	0.132	0.42	0.170	NA	NA	NA	NA	8.2E-13	0.357 (0.259, 0.455)	0.390

The frequency of rs115242848 [T] in the listed populations is: Iceland = 1.22%, Denmark = 1.34%, Australia = 0.87%, Hong Kong and Korea = not polymorphic.

Supplementary table 16: Association of rs115242848 [T] in *EN1* with osteoporotic fractures at different skeletal sites in the discovery and individual replication samples

P value	OR	*P* value	OR	*P* value	OR	*P* value	OR	*P* value	OR	*P* value	OR	*P* het
All fractures												
**Ice (7,836/261,563)**		**DK (1,500/1,284)**		**AUS (482/506)**		**HK (400/826)**		**KOR (171/343)**		**All sets combined (10,389/264,522)**		
0.015	0.66	0.022	0.53	0.26	0.55	NA	NA	NA	NA	0.00054	0.61 (0.46, 0.81)	0.79
Vertebral fracture												
**Ice (263/176,455)**		**DK (824/1,284)**		**AUS (189/506)**		**HK**		**KOR (106/343)**		**All sets combined (1,382/178,588)**		
0.056	0.31	0.15	0.66	0.58	0.60	NA	NA	NA	NA	0.028	0.58 (0.35, 0.94)	0.54
Hip fracture												
**Ice (6,239/115,587)**		**DK (79/1,284)**		**AUS (126/506)**		**HK**		**KOR**		**All sets combined (6,444/117,377)**		
0.070	0.81	0.72	0.89	0.75	0.61	NA	NA	NA	NA	0.065	0.82 (0.67, 1.01)	0.95
Forearm fracture												
**Ice (1,102/196,718)**		**DK (569/1,284)**		**AUS (113/506)**		**HK**		**KOR**		**All sets combined (1,784/198,508)**		
0.10	0.65	0.14	0.60	0.49	0.34	NA	NA	NA	NA	0.022	0.63 (0.42, 0.93)	0.91

The frequency of rs115242848 [T] in the listed populations is: Iceland = 1.22%, Denmark = 1.34%, Australia = 0.87%, Hong Kong and Korea = not polymorphic.
